# The role of ion channels in the hypoxia-induced aggressiveness of glioblastoma

**DOI:** 10.3389/fncel.2014.00467

**Published:** 2015-01-15

**Authors:** Luigi Sforna, Marta Cenciarini, Silvia Belia, Maria Cristina D’Adamo, Mauro Pessia, Fabio Franciolini, Luigi Catacuzzeno

**Affiliations:** ^1^Department of Chemistry, Biology and Biotechnology, University of PerugiaPerugia, Italy; ^2^Faculty of Medicine, Section of Physiology and Biochemistry, Department of Experimental Medicine, University of PerugiaPerugia, Italy

**Keywords:** hypoxia, glioblastoma multiforme (GBM), ion channels, aggressiveness, cancer

## Abstract

The malignancy of glioblastoma multiform (GBM), the most common and aggressive form of human brain tumors, strongly correlates with the presence of hypoxic areas, but the mechanisms controlling the hypoxia-induced aggressiveness are still unclear. GBM cells express a number of ion channels whose activity supports cell volume changes and increases in the cytosolic Ca^2+^ concentration, ultimately leading to cell proliferation, migration or death. In several cell types it has previously been shown that low oxygen levels regulate the expression and activity of these channels, and more recent data indicate that this also occurs in GBM cells. Based on these findings, it may be hypothesized that the modulation of ion channel activity or expression by the hypoxic environment may participate in the acquisition of the aggressive phenotype observed in GBM cells residing in a hypoxic environment. If this hypothesis will be confirmed, the use of available ion channels modulators may be considered for implementing novel therapeutic strategies against these tumors.

## Introduction

Glioma is a major tumor involving glial cells in the central nervous system, and accounting for 35–50% of intracranial tumors in adults. Among them grade IV glioblastoma multiform (GBM) has the highest incidence and shortest patient survival, with approximately three cases per 100,000 person-years and median survival of only approximately 10 months. The longest survival is achieved in patients who undergo resection followed by radiotherapy and chemotherapy, yet the median survival with this combined treatment is still only 20 months (Holland, 2001). Increasing malignancy of gliomas strongly correlates with insufficient blood supply, hypoxic areas and necrotic formations. Many hypoxic regions are found in GBM, including a central large necrotic core and multiple thrombotic foci surrounded by pseudopalisading cells migrating away towards more oxygenated areas (Rong et al., 2006; Amberger-Murphy, 2009). Hypoxia represents a major driving force for the development of tumor aggressiveness through increased invasion, resistance to apoptosis, chemo and radioresistance, and tumorigenic cancer stem cells development (Yang et al., 2012). While few of these effects are mediated acutely and “directly” by the hypoxic condition, in most cases hypoxia acts by gene expression changes promoted by hypoxia-induced factors (HIFs; Yang et al., 2012).

GBM cells express a number of ion channels, membrane proteins that allow the selective and controlled passage of ions along their electrochemical gradient. Most of them have a deregulated expression in GBM as compared to normal glial cells, and the resulting alteration in membrane ion flux has important consequences in the acquisition of the features typical of the transformed cell. For example, the coordinated activity of the upregulated K^+^ and Cl^−^ channels generates transmembrane fluxes of the respective ions supporting membrane potential changes as well as cell volume changes promoted by the osmotically driven water flux. Volume changes are in turn strongly required for a number of basic cell functions, such as cell division and growth, migration and death (Akita and Okada, 2014). Likewise, Ca^2+^-selective ion channels, important modulators of the Ca^2+^-dependent enzymes, are also often upregulated in GBM, where they promote a number of important cell functions (Santoni et al., 2012). Thus, ion channels may control most of the cell functions also regulated by a hypoxic environment and may play important roles in the mechanisms leading to hypoxia-modulated GBM aggressiveness.

In several tissues, low oxygen levels regulate the expression or activity of many ion channels, including those controlling the aggressiveness of GBM (Shimoda and Polak, 2011). Since the initial description of a hypoxia-regulated ion channel (López-Barneo et al., 1988), numerous studies have been carried out indicating that a variety of ion channels are sensitive to oxygen, exhibiting changes in channel activity with acute hypoxia and alterations in channel expression with prolonged hypoxic challenge (chronic hypoxia).

Based on these observations we carried out a literature survey aimed at addressing the following two questions: (i) Are there GBM ion channels involved in the control of certain aspects of cell aggressiveness that are also regulated by hypoxia? (ii) For these specific channels, do experiments performed in GBM or in other preparations have highlighted a hypoxic-mediated modulation of their expression or activity? This undertaking has been made bearing in mind the rational that ion channels would be involved in the hypoxia-induced aggressiveness of GBM.

## Chemoresistance

Among the chemotherapics most used in the treatment of GBM there are temozolomide (TMZ), carmustine (bis-chloroethylnitrosourea; BCNU) and doxorubicin (DOXO), all DNA alkylating agents causing DNA damage and cell death. Although the use of chemotherapics plays an important role in the combined treatment of GBM, it remains a challenge because of tumor chemoresistance (Haar et al., 2012). Among the many factors contributing to GBM chemoresistance is hypoxia, both with direct and indirect mechanisms. An obvious reason for this resistance is that hypoxic areas are often relatively far from blood vessels, preventing the chemotherapic agent from reaching its target (Vaupel et al., 2001). Another protective factor is the non proliferative nature of hypoxic cells, that makes them intrinsically resistant to the anticancer agents acting preferentially on cycling cells (Oliver et al., 2009). More detailed molecular research also suggests that among the targets of HIF-1 is MDR1, the gene encoding for P-glycoprotein, the drug transporter that strongly contributes to chemoresistance by a drug efflux activity (Comerford et al., 2002).

### Gap junctions

Several studies show an involvement in GBM chemoresistance for gap junctions, intercellular ion channels that allow the passage of small ions and molecules between neighboring cells (Simon and Goodenough, 1998; Goldberg et al., 1999; Fry et al., 2001). In astrocytes and astrocytoma cells gap junctions are formed by the connexin 43 (Cx43) subunits. It has been recently shown that increasing the level of Cx43 in human LN18 and LN229 glioma cells enhances resistance to TMZ, while knockdown of Cx43 sensitizes them to TMZ, demonstrating a fundamental role of gap junctions in the responsiveness to chemotherapics (Gielen et al., 2013). In these cells Cx43 alters mitochondrial apoptotic pathways by regulating the level of Bax2 and Bcl-2, as well as Cyt C release from the mitochondria following TMZ treatment (Gielen et al., 2013). In addition to its recognized role as channel protein, Cx43 has a “non-channel” mechanism of action involved in intercellular signaling (Goodenough and Paul, 2003; Naus and Laird, 2010), and this mode of action is thought to reverse the oncogenicity of human GBM cells (Huang et al., 1998). Interestingly, the Cx43-dependent TMZ resistance was found to depend on both its channel-dependent and -independent mechanisms, as demonstrated by using Cx43 channel-defective mutants (Gielen et al., 2013). Although the effects of hypoxia on GBM gap junctions have not been investigated, several studies in other preparations point to a possible modulatory role. In cardiac tissue and MDCK epithelial cells expressing Cx43, hypoxia rapidly activates Akt, which leads to Cx43 phosphorylation and to larger gap junctions (Dunn and Lampe, 2014). Also in human mesenchymal stem cells Cx43 expression is increased by hypoxia (Grayson et al., 2007). Conversely in rat H9C2 and HL-1 cardiomyocytes hypoxia promotes Cx43 degradation (Severino et al., 2012; Wu et al., 2013). In cultured astrocytes Cx43 underwent dephosphorylation 30 min after hypoxia, and this was preceded by a strong reduction in gap junctional intercellular communication (Li and Nagy, 2000). Based on this evidence, it may be hypothesized that Cx43 participate in the hypoxia-induced chemoresistance observed in GBM.

### Cl^−^ channels

A number of different Cl^−^ channels have been reported to be expressed in GBM cells and to be implicated in several cell functions, including chemoresistance (Sontheimer, 2004; Catacuzzeno et al., 2011, 2014; Cuddapah and Sontheimer, 2011). It was found that a BCNU-resistant subpopulation of GBM cells upregulated the ClC-1 intracellular Cl^−^ channel, whereas the unspecific Cl^−^ channel inhibitor DIDS synergistically augmented the apoptotic efficacy of BCNU (Kang and Kang, 2008). In addition, specific inhibition of ClC-3 expression by siRNA-mediated knockdown sensitized U251 cells to cisplatin-mediated cell death, through the downregulation of phosphorylated Akt (Su et al., 2013). Again, although no evidence is present in GBM cells for a hypoxia-induced modulation of Cl^−^ channels, in other preparations this modulation was found. For example, chronic hypoxia upregulates Ca^2+^-activated Cl^−^ channels in vascular smooth muscle cells (Sun et al., 2012), while acute hypoxia inhibited the swelling-activated Cl^−^ current (I_Cl,Swell_) in cerebellar granule neurons (Patel et al., 1998). These data suggest a possible hypoxia-mediated modulation of Cl^−^ channels also in GBM, that can have a role in the hypoxia-induced chemoresistance.

## Migration/Invasion

GBM cells often invade adjacent normal brain tissue, and this infiltrative behavior makes complete surgical resection impossible, and tumor recurrences most frequent (Claes et al., 2007; Lefranc et al., 2009). Several studies have shown that hypoxia is a major promoter of GBM invasion. Under hypoxic conditions GBM cells increase both their bi-dimensional migratory ability, as tested by wound-healing assay, and their invasiveness on matrigel-coated transwell (Shen et al., 2013; Zhang et al., 2013). Both these types of hypoxia-induced migratory abilities were mediated by HIF-1, as demonstrated through knock-down experiments (Méndez et al., 2010; Esencay et al., 2013; Fujimura et al., 2013).

### Cl^−^ channels

Several studies show that GBM cells over-express the Cl^−^ channels ClC-3 and the Ca^2+^-activated K^+^ channels KCa1.1 and KCa3.1, and their coordinated activity, promoting K^+^ and Cl^−^ transmembrane fluxes, supports cell volume and membrane potential changes necessary for GBM cell migration and invasion (Catacuzzeno et al., 2012; Turner and Sontheimer, 2014). ClC-3 channels have been shown to play an important role in cell migration and invasion (Olsen et al., 2003; Ernest et al., 2005; McFerrin and Sontheimer, 2006), and found to be localized in invadopodia where they form protein complexes with other important modulators of cell invasion, such as matrix metalloprotease-2 and aquaporin-4 (Deshane et al., 2003). Given the previously mentioned evidence for a hypoxia-induced modulation of Cl^−^ channels, it may be hypothesized that a change in Cl^−^ channel expression or activity may have a role in the hypoxia-induced GBM invasiveness.

### Ca^2+^-activated K^+^ channels

KCa1.1 and KCa3.1 channels have also been shown to be required by several pro-migratory signals (Sontheimer, 2008; Sciaccaluga et al., 2010; Catacuzzeno et al., 2011). KCa1.1 channels are expressed as a particular splicing isoform in GBM cells (the glioma BK, gBK; Liu et al., 2002), and are implicated in GBM invasion (Bordey et al., 2000; Wondergem and Bartley, 2009; Cuddapah and Sontheimer, 2011; Steinle et al., 2011). KCa3.1 channels are most important in GBM as they are virtually not expressed in normal, differentiated glial cells. Their presence could thus be exploited as an important diagnostic tool, and their pharmacological targeting could well represent a potential therapeutic strategy for this tumor (Catacuzzeno et al., 2012). It has been found that several recognized pro-migratory signals, such as fetal calf serum and SDF-1, activate KCa3.1 channels, leading to GBM cell migration (Sciaccaluga et al., 2010; Catacuzzeno et al., 2011).

Several studies show that KCa1.1 and KCa3.1 channels can be modulated by chronic hypoxia. In human pulmonary smooth muscle cells hypoxia increases the expression of both the KCa1.1 α and the β1 subunits through a HIF-1α-mediated mechanism (Resnik et al., 2006; Ahn et al., 2012). Conversely in human podocytes hypoxia causes a significant reduction in KCa1.1 channel currents, by an increase of the KCa1.1 β4 subunit, that causes a shift of the channel activation range toward more depolarized voltages (Zhang et al., 2012a). In alveolar epithelial A549 cells and rat carotid body type I cells KCa1.1 channels appear also sensitive to acute hypoxia (Riesco-Fagundo et al., 2001; Jovanović et al., 2003). In addition hemoxygenase-2 (HO-2) has been found to be part of the KCa1.1 channel complex, and modulate channel activity depending on the oxygen levels (Williams et al., 2004). Hypoxia can also directly inhibit specific KCa1.1 channel splicing isoforms containing a cysteine-serin (CS) motif in its C-terminal segment (McCartney et al., 2005), a feature also displayed by the gBK isoform (Liu et al., 2002). However, a recent study performed in GBM cells has shown that acute hypoxia activates KCa1.1 channels residing in the mitochondrial membrane, while no effect was observed on plasmamembrane KCa1.1 channels (Gu et al., 2014). The effects of chronic hypoxia on the expression or properties of GBM KCa1.1 channels, and whether this modulation has a role in the hypoxia-induced invasive phenotype, remain to be tested. Finally, in intestinal epithelial cells chemical hypoxia modulates the basolateral KCa3.1 activity (Loganathan et al., 2011).

### TRPC6 channels

One of the few cases where a role of ion channels in the hypoxia-induced aggressiveness has been clearly demonstrated is represented by the Ca^2+^-permeable transient receptor potential 6 (TRPC6). In GBM cells hypoxia increases Notch1 activation, which induces the expression of TRPC6, resulting in an increased basal intracellular Ca^2+^ concentration and activation of the calcineurin-nuclear factor of activated T-cell (NFAT) pathway (Chigurupati et al., 2010). The hypoxia-induced TRPC6 upregulation is required for the development of the aggressive phenotype as TRPC6 knock-down inhibited glioma growth, invasion, and angiogenesis. Finally, expression of TRPC6 was elevated also in GBM specimens in comparison with normal tissues (Chigurupati et al., 2010).

### T-type Ca^2+^ channels

U87 GBM cells express all three α subunits of T-type Ca^2+^ channels, a class of Ca^2+^ permeable low-voltage activated channels that open in response to small depolarization of the membrane, and whose activity increases the transwell migratory ability of these cells (Zhang et al., 2012b). Notably, hypoxia modulates T-type Ca^2+^ channels in many different cell types. In PC12 cells and adult chromaffin cells, the α1 subunit mRNA expression and the number of functional T-type Ca^2+^ channels are upregulated by hypoxia (Del Toro et al., 2003; Carabelli et al., 2007). It may thus be possible that a hypoxia-induced upregulation of T-type Ca^2+^ channels in GBM cells participates in enhancing the invasive potential of these cells under hypoxic conditions.

### Store-operated Ca^2+^ channels

A role in GBM cell migration has also been found for the store-operated Ca^2+^ channels, Ca^2+^ permeable channels whose activity is controlled by the filling level of the intracellular Ca^2+^ stores. In two different primary cultures from GBM biopsies knock-down of the STIM1/ORAI proteins encoding for the store-operated Ca^2+^ channels strongly reduced the serum-induced chemotactic migration through transwell membranes (Motiani et al., 2013). Interestingly, in pulmonary artery smooth muscle cells, acute hypoxia activates the store-operated Ca^2+^ entry (Peng et al., 2013), and in the distal intrapulmonary arteries chronic hypoxia upregulates the expression of STIM1 (Hou et al., 2013). In addition Wilms tumor suppressor 1, a recognized inhibitor of STIM1 expression (Ritchie et al., 2010), has been identified as a target of HIF-1 (Scholz and Kirschner, 2011). All these data point to the suggestion that a hypoxia-induced modulation of the store-operated Ca^2+^ entry may contribute in promoting the invasion of GBM cells.

## Radioresistance

Radiation therapy impairs the growth and survival of tumor cells mainly by causing double strand breaks in the DNA backbone. It is included, as adjuvant therapy, in the surgical and chemotherapic treatments of GBM. However, outcomes are poor, and GBM remains an incurable disease with the majority of recurrences and progression within the radiation treatment field (Alexander et al., 2013). Tumor hypoxia represents a severe problem for radiation therapy, as radiosensitivity rapidly decreases when the O_2_ partial pressure is less than 25–30 mmHg. Since in low oxygen the DNA damage speeds up, the radiotherapic dose required to achieve the same biologic effect is markedly higher under hypoxia (Gray et al., 1953). Several lines of evidence indicate that hypoxia is involved in the transcriptional modulation of genes that control cell growth and resistance to apoptotic cell death, and the selection of more resistant cell clones, and all these actions may have a major impact on radioresistance (Vaupel et al., 2001; Vaupel, 2004; Amberger-Murphy, 2009; Jensen, 2009). Despite a full course of radiotherapy, up to 90% of GBM relapse in proximity of areas with high HIF-1 expression (Brat and Van Meir, 2004; Rong et al., 2006). In addition the intensity of hypoxia in GBM before radiotherapy is strongly associated with enhanced tumor progression and decreased patient survival (Spence et al., 2008). Recent studies, both in cultured GBM cells and in xerograph models, show that cycling (intermittent) hypoxia induces HIF-1 upregulation, nox4-mediated ROS production and increased radioresistance (Hsieh et al., 2010, 2012).

### KCa1.1 channel

A well-known form of radioresistance is the promotion of tumor cell invasiveness by irradiation (Camphausen et al., 2001; Wild-Bode et al., 2001; Qian et al., 2002; Cordes et al., 2003; Park et al., 2006). Interestingly, it has been found that irradiation of GBM cells raises the intracellular Ca^2+^ concentration and stimulates KCa1.1 channel activity, which result in the activation of CaMKII and enhanced cell migration (Steinle et al., 2011). Notably, both CaMKII activation and the enhanced migration were abolished by the KCa1.1 channel inhibitor paxilline (Steinle et al., 2011). As several studies have shown that in GBM cells and in other tissues KCa1.1 channels are modulated by hypoxia, these results suggest that the hypoxia-mediated modulation of KCa1.1 channels may participate to the hypoxia-induced GBM radioresistance.

## Resistance to apoptosis

It has long been known that hypoxia increases the anti-apoptotic potential of tumor cells by modulating the expression and activity of the molecules involved in the apoptotic pathways (Harris, 2002). In GBM cells held under hypoxia BNIP3, a pro-apoptotic member of the Bcl-2 family, becomes unable to translocate to the cytoplasm and mediate hypoxia-induced cell death, thus favoring cell survival through the loss of apoptotic potential (Burton et al., 2006). Moreover, in GBM cells chronic hypoxia induces Bad phosphorylation and prevents its binding to Bcl-XL, promoting survival of GBM cells (Merighi et al., 2007).

### Cl^−^ and Ca^2+^-activated K^+^ channels

Several ion channels expressed in GBM cells are required for the apoptotic process. An early hallmark in apoptosis is the cellular condensation, termed apoptotic volume decrease (AVD). K^+^ and Cl^−^ effluxes have an essential role in this mechanism as they establish the driving force for cytoplasmic water efflux from the cell. It has been shown that AVD in D54-MG glioma cells is dependent on a DIDS-sensitive chloride conductance (Ernest et al., 2008), and a K^+^ efflux mediated by Ca^2+^-activated K^+^ channels, KCa3.1 and KCa1.1 (McFerrin et al., 2012). These channels play differential roles in apoptosis: KCa3.1 mediates AVD in response to the activation of the intrinsic pathway, consisting in the mitochondrial disruption by various stressors, whereas KCa1.1 is engaged by the extrinsic pathway, involving the stimulation by extracellular ligands. The involvement of these two channels in different apoptotic pathways is explained by the different temporal Ca^2+^ profiles during induction of apoptosis (McFerrin et al., 2012). We have already mentioned on the evidence of the hypoxia-induced modulation of KCa1.1, KCa3.1, and Cl^−^ channels. If these findings will be confirmed in GBM cells, a role of ion channels in the hypoxia-induced resistance to cell death becomes likely.

### TRPV1 and T-type Ca^2+^ channels

In U373 glioma cell line, capsaicin stimulation of TRPV1 channels induced a rapid Ca^2+^ influx, DNA fragmentation, externalization of phosphatidilserine, mitochondrial transmembrane potential dissipation, and activation of caspase 3 and p38 MAPK (Amantini et al., 2007). In addition in GBM cell lines T-type Ca^2+^ channels are over-expressed and their activity is correlated with tumor proliferation and progression. Valerie et al. (2013) showed that in glioma cell lines the pharmacological inhibition or siRNA-mediated knock-down of T-type Ca^2+^ channel leads to apoptotic cell death by inhibiting mTOR/Akt pro-survival signaling pathways. We have already discussed the ability of hypoxia to modulate T-type Ca^2+^ channels. As for TRPV1 channel, in pulmonary artery smooth muscle cells, the TRPV1 gene and protein are upregulated by chronic hypoxia (Wang et al., 2008). Both TRPV1 and T-type Ca^2+^ channels may thus potentially be involved in the hypoxia-induced resistance to apoptosis in GBM cells.

## Conclusions

In this perspective we have taken several examples of GBM aggressiveness and showed how they strictly relate to hypoxia and ion channels. Namely, we provided evidence that hypoxia promotes GBM aggressive phenotypes, that several types of ion channel are deeply involved in driving GBM aggressiveness, and that hypoxia heavily modulates these very channels promoting GBM aggressiveness. These lines of evidence are in our view more than a circumstantial coincidence. On the contrary they appear strong indications to put forward the working hypothesis that hypoxia promotes the various forms of GBM aggressiveness through mechanisms involving also ion channels as relevant players. This working hypothesis could be taken as a basis to direct future research.

## Conflict of interest statement

The authors declare that the research was conducted in the absence of any commercial or financial relationships that could be construed as a potential conflict of interest.
